# Modulation of Apoptotic Pathways of Macrophages by Surface-Functionalized Multi-Walled Carbon Nanotubes

**DOI:** 10.1371/journal.pone.0065756

**Published:** 2013-06-06

**Authors:** Yuanqin Jiang, Honggang Zhang, Yange Wang, Min Chen, Shefang Ye, Zhenqing Hou, Lei Ren

**Affiliations:** 1 Department of Biomaterials, College of Materials, Xiamen University, Xiamen, China; 2 Department of E. N. T., Chenggong Hospital of Xiamen University, the 174^th^ Hospital of People’s Liberation Army, Xianmen, China; Argonne National Laboratory, United States of America

## Abstract

Biomedical applications of carbon nanotubes (CNTs) often involve improving their hydrophilicity and dispersion in biological media by modifying them through noncovalent or covalent functionalization. However, the potential adverse effects of surface-functionalized CNTs have not been well characterized. In this study, we functionalized multi-walled CNTs (MWCNTs) via carboxylation, to produce MWCNTs-COOH, and via poly (ethylene glycol) linking, to produce MWCNTs-PEG. We used these functionalized MWCNTs to study the effect of surface functionalization on MWCNTs-induced toxicity to macrophages, and elucidate the underlying mechanisms of action. Our results revealed that MWCNTs-PEG were less cytotoxic and were associated with less apoptotic cell death of macrophages than MWCNTs-COOH. Additionally, MWCNTs-PEG induced less generation of reactive oxygen species (ROS) involving less activation of NADPH oxidase compared with MWCNTs-COOH, as evidenced by membrane translocation of p47^phox^ and p67^phox^ in macrophages. The less cytotoxic and apoptotic effect of MWCNTs-PEG compared with MWCNTs-COOH resulted from the lower cellular uptake of MWCNTs-PEG, which resulted in less activation of oxidative stress-responsive pathways, such as p38 mitogen-activated protein kinases (MAPK) and nuclear factor (NF)-κB. These results demonstrate that surface functionalization of CNTs may alter ROS-mediated cytotoxic and apoptotic response by modulating apoptotic signaling pathways. Our study thus provides new insights into the molecular basis for the surface properties affecting CNTs toxicity.

## Introduction

Motivated by their various novel properties, engineered nanomaterials have been increasingly considered as biomaterials for biomedical and pharmaceutical applications [1]. Among these nanotechnology-derived nanomaterials, carbon nanotubes (CNTs) have stimulated a great interest because of their unique properties that enable them to be used as versatile platforms for a variety of biomedical applications, including protein and peptide transport [2], drug and gene delivery [3], medical imaging [4], and cancer targeting and therapeutics [5].

Although CNTs look promising, they are also plagued with uncertainty about their potentially hazardous effects on human health due to their nanosized scale, permeability through organic barriers, and asbestos-like fiber shape [6]. Research has shown that the toxicity of CNTs depends on their physicochemical properties, including their purity, surface chemistry, dimensions, and surface area [7], [8]. For example, previous studies have indicated that pure multi-walled CNTs (MWCNTs) can injure the plasma membrane of human macrophages [9] and rat astrocytes [10]. These observations suggest that pure CNTs without surface modification are cytotoxic to certain mammalian cells. Therefore, strategies for surface functionalization, including covalent and noncovalent functionalization, have recently increased in popularity, since functionalized MWCNTs (*f*-MWCNTs) are generally considered more biocompatible than pure CNTs due to improved hydrophilicity and dispersion in biological media [11]. Previous studies have shown that carboxylated [12], pluronic-coated [13], taurine- [14], and polystyrene-functionalized CNTs [15] induce less cytokine production, pulmonary inflammation, and fibrosis than pure CNTs. Their conclusions support the notion that surface chemistry is one of the primary determinants of CNTs toxicity. With a wide range of biomedical applications, different types of surface-modified CNTs should be systematically evaluated in terms of cellular uptake and cytotoxicity.

Poly(ethylene glycol)-functionalized (PEGylated) CNTs have received enormous attention in various biomedical applications, since PEGylated CNTs exhibit excellent individual dispersion and stability in various biological solutions, a relatively long life in circulating blood, and low uptake in the reticuloendothelial system i*n vivo* [11]. Recent studies assessed the effects of surface PEGylation of CNTs on long-term hepatotoxicity *in vivo* [16], oxidative stress-mediated toxicity [17], and proinflammatory response *in vitro* [18]. The results of these studies collectively suggest that surface functionalization with PEG is critical to the toxic behaviors of CNTs. Recently, apoptosis has been suggested as a potential event related to toxic effects induced by various nanomaterials, including CNTs [19]. However, the effects of surface functionalization of CNTs on apoptosis *in vitro* remain largely unknown.

In this study, we prepared PEGylated MWCNTs (MWCNTs-PEG) and carboxylated MWCNTs (MWCNTs-COOH) and investigated their ability to induce apoptosis in RAW 264.7 macrophage cells. We found that MWCNTs-PEG were less cytotoxic and associated with less apoptotic cell death compared with MWCNTs-COOH, thus demonstrating the role of surface properties on CNTs toxicity. The mechanism of these effects involves differences in cellular uptake of *f*-MWCNTs and apoptotic pathway activation in macrophages.

## Materials and Methods

### Preparation and Characterization of MWCNTs-COOH and MWCNTs-PEG

MWCNTs synthesized via chemical vapor deposition were purchased from Nanotech Port Co. Ltd., (Shenzhen, China). The MWCNTs were purified through H_2_SO_4_/HNO_3_ (3∶1) treatment as described in our previous work [20]. This process resulted in the generation of carboxylic (-COOH) groups on the surface of the nanotubes. To PEGylate the MWCNTs, carboxylated MWCNTs (30 mg) were suspended in 3 mL thionyl chloride for 12 h, after which unreacted thionyl chloride was removed by centrifugation. The sample was then mixed with PEG (MW 1500, Fluka, USA) and stirred at 100°C for 24 h under nitrogen protection. The soluble fraction containing the MWCNTs-PEG was separated from the insoluble residue via centrifugation at ∼1400 × g for 15 min. The excess free PEG was removed via dialysis tubing (MWCO ∼12,000) against deionized water for 3 days to obtain the MWCNTs-PEG sample.

A transmission electron microscope (TEM) (Tecnai F30, Philips-FEI) was used to visualize the morphology of *f*-MWCNTs. A Fourier transform infrared (FT-IR) spectroscope (Nicolet Avatar 360 FT-IR) was used to analyze the surface chemistry of *f*-MWCNTs. Thermogravimetry analysis (TGA) was performed using a TGA analyzer (Netzsch STA 409 EP) at a heating rate of 20°C/min under nitrogen atmosphere. Additional characterization of *f*-MWCNTs included X-ray photoelectron spectroscopy (XPS, K-Alpha, Thermofisher), inductively coupled plasma mass spectrometry (ICP-MS) (HP 4500), Zetasizer Nano ZS (Malvern, UK), and Brunauer-Emmer-Teller (BET) surface area analysis (Micromeritics TriStar 3000).

MWCNTs-COOH and MWCNTs-PEG were freshly suspended in RPMI1640 medium (Gibco, Grand Island, NY, USA) supplemented with 10% FBS according to a previously described method [21]. A stable suspension of MWCNTs-COOH and MWCNTs-PEG in medium with no dispersing reagent was obtained in this way and used immediately. In addition, carbon black particles with an average diameter of 21 nm (Degussa, Frankfurt, Germany) were used as a control to compare the effects of MWCNTs-COOH and MWCNTs-PEG on macrophages.

### Macrophage Isolation and Cell Culture

Female BALB/c mice (6∼8-week old; Animal Research Laboratory, Xiamen University) received intraperitoneal injections of 1.5 mL of sterile 4% thioglycollate solution. Seven days later, peritoneal macrophages were collected from their peritoneal cavities, as previously described [22]. All animal procedures performed in this work were in accordance with the recommendations in the Guide for the Care and Use of Laboratory Animals of the National Institutes of Health. The protocol was approved by the Committee on the Ethics of Animal Experiments of the University of Xiamen (Approval Number: 2011058). After washing with RPMI 1640 medium containing 2% FBS, peritoneal macrophages (1×10^6^ cells/mL) were plated in 100 mm tissue culture dishes for 4 h at 37°C in a 5% CO_2_, humidified atmosphere. RAW 264.7 cells (American Type Culture Collection, ATCC) and peritoneal macrophages were cultured in RPMI1640 medium supplemented with 10% heat-inactivated FBS, glutamine, and antibiotics (penicillin and streptomycin) at 37°C under 5% CO_2_.

### Cell Viability Assay

The WST-1 assay was used according to the manufacturer’s instructions (Roche, Penzberg, Germany) to determine the viability and proliferation of RAW 264.7 cells and peritoneal macrophages after treatment with the *f*-MWCNT suspension. Cells were seeded in 96-well tissue culture plates at the density of 1×10^5^ cells per well and incubated overnight before MWCNTs-COOH or MWCNTs-PEG was added at various concentrations. After treatment for 24 h, the plates were washed twice with culture medium. WST-1 (diluted with HBSS, 1∶20 v/v) was then added to the cells, which were incubated for another 2 h. The absorbance at 450 nm was recorded on a microplate spectrophotometer (Bio-rad Model 680, USA). The results were reported relative to the untreated control.

### Cell Uptake Study

Increase in nanoparticle uptake leads to an increase in cellular granularity. We quantified this increase measuring the light side-scatter (SSC) intensity via flow cytometry (Beckman-Coulter Epics XL, Miami, USA) according to our previously described method [21]. Fluorescein isothocyanate (FITC)-BSA is efficiently absorbed by MWCNTs through π-π stacking and electrostatic interactions [23]. Therefore, FITC was employed as the fluorescent marker to evaluate the cellular uptake and distribution of *f*-MWCNTs as previously described [23], [24].

### Apoptosis Assay

The terminal deoxynucleotidyl transferase dUTP nick-end labeling (TUNEL) assay (Invitrogen, Carlsbad, CA) was performed after washing paraformaldehyde-fixed cells on a coverslip once with phosphate-buffered saline (PBS) and subsequently permeabilizing them with 0.1% Triton X-100 in PBS. The fixed and permeabilized cells were incubated first with 100 µL/well TUNEL reaction solution containing a nucleotide mixture and terminal deoxynucleotidyl transferase (TdT) for 1 h, then 1 µg/mL of DAPI for 15 min at 37°C. The cells were then washed with PBS and examined under a fluorescence microscope (Nikon Eclipse 80i, Tokyo, Japan). For the annexin V assay, coverslips were washed with PBS and incubated for 15 min at room temperature with a solution of annexin-V-FLUOS and propidium iodide (Roche, Mannheim, Germany). The cells were then washed twice with PBS and analyzed by flow cytometry. For agarose gel electrophoresis, the characteristic ladder pattern of DNA breakage was analyzed by the Apoptosis DNA Ladder Detection Kit according to the manufacturer’s instruction (Beyotime Biotech, Haimen, China). The gel was photographed under a UV transilluminator (GeneGenius Super 12, Syngene, UK). Fragmented DNA, shown as a DNA ladder in the gel, indicated apoptotic cell death.

### Caspase Activity Assay

Following treatment with the *f*-MWCNTs, cells were measured for caspase-3, −8, and −9 activities using a Caspase-Glo assay kit (Promega, Madison USA). Briefly, the plates containing cells were removed from the incubator and allowed to equilibrate to room temperature for 30 min. Then, 100 µL of Caspase-Glo reagent was added to each well and gently mixed using a plate shaker at 300–500 rpm for 30 s. The plate was then incubated at room temperature for 2 h. The luminescence of each sample was measured with a fluorescent microplate reader (BIOTEK, FL-600).

### Immunofluorescence Staining

RAW 264.7 cells were fixed with paraformaldehyde, permeabilized with 0.5% Triton X-100 in PBS, and then incubated with blocking buffer (PBS, 5% goat serum, and 0.3% Triton X-100) for 30 min. The cells were then labeled with antibodies against p47^phox^ and p65 (Santa Cruz Biotechnology, Cruz, CA) in blocking buffer at 4°C overnight, followed by incubation with a FITC-conjugated secondary antibody (Santa Cruz Biotechnology, Cruz, CA). Thereafter, cells were nuclear-stained via 15-min incubation in a blocking solution containing 0.25 mg/mL DAPI or Hoechst. Fluorescent-labeled cells were imaged with a fluorescent microscope (Leica DMR, Germany).

### Measurement of Intracellular Reactive Oxygen Species (ROS) Generation

Intracellular ROS was measured using peroxide-sensitive fluorescent probe 2′, 7′-dichlorofluorescein diacetate (DCFH-DA; Molecular Probes, Eugene, OR) and flow cytometry. DCFH-DA enters the cells via passive diffusion where it reacts with ROS resulting in the formation of the highly fluorescent compound, dichlorofluorescein (DCF) [25]. In addition, generation of superoxide radicals was examined using chemical probes of dihydroethidium (DHE; Molecular Probes, Eugene, OR). DHE, which is nonfluorescent and membrane-permeable, causes the release of membrane impermeable ethidium cations by its interaction with superoxide [21]. After treatment with the MWCNT samples in the absence or presence of diphenylene iodium (DPI), an inhibitor of NADPH oxidase, or N-acetylcysteine (NAC), a ROS scavenger, cells were washed, incubated with 10 µM H_2_DCFDA or 5 µM DHE for 30 min at 37°C, and then analyzed using a flow cytometer (Beckman-Coulter Epics XL, Miami, USA) or examined with a fluorescent microscope (Leica DMR, Germany). In each experiment, a positive control for DCF and ethidium detection was performed by incubating macrophages with H_2_O_2_ (1 mM).

### NADPH Oxidase Activity Assay

Following incubation with various MWCNT treatments, cells were pelleted by centrifugation at 3000 g for 10 min. Cells were sonicated in a buffer containing 50 mmol/L phosphate buffer (pH 7.0), 1 mmol/L EDTA, 1 mmol/L PMSF, and 1 mmol/L leupeptin on ice. Cell lysates were incubated for 2 min with 5 µmol/L lucigenin in 50 mmol/L phosphate buffer. NADPH substrate (100 µmol/L) was added to the reaction mixture and the chemiluminescent signal was measured every 15 s for 3 min using a Lumbat LB 9507 luminometer (Berthold Technologies, Bad Wildbad, Germany).

### Mitochondrial Membrane Potential (Δ*Ψ*m) Assay

The mitochondrial membrane potential (Δ*Ψ*m) was assessed using the lipophilic fluorochrome, JC-1 (Molecular Probes, Eugene, OR). Briefly, cells were treated with 75 µg/mL of either MWCNTs-COOH or MWCNTs-PEG for the previously mentioned time periods. After treatment, cells were trypsinized and resuspended in 0.5 mL of PBS containing 10 µg/mL of JC-1. After incubation for 10 min at 37°C, cells were immediately centrifuged to remove the supernatant. Cell pellets were resuspended in PBS and then analyzed by flow cytometry. The percentage of green fluorescence from the JC-1 monomers was used to represent the cells that lost Δ*Ψ*m.

### Membrane Fractionation

Following treatment with the MWCNT samples, cells were harvested and sonicated on ice in a buffer containing 100 mM KCl, 3 mM NaCl 3.5 mM MgCl_2_, 10 mM HEPES, 1 mM EGTA, 10 µg/mL pepstatin, 10 µg/mL leupeptin, and 0.5 mM PMSF. Cell lysates were centrifuged for 5 min at 500 × g at 4°C. Resulting supernatants were collected and centrifuged for 20 min at 13,000 × g at 4°C. The pellets were further centrifuged for 1 h at 100,000 × g at 4°C, and the resulting supernatants and pellets were designated as the cytosolic and plasma membrane fractions, respectively [26]. Membranes were washed in the same buffer, quantified, and resuspended in Laemmli sample buffer, before western blot analysis.

### Electrophoretic Mobility Shift Assay (EMSA)

Nuclear extracts from cells incubated with either MWCNTs-COOH or MWCNTs-PEG were prepared as described previously [27]. EMSA were performed using a double-stranded oligonucleotide (5′-AGTTGAGGGG ACTTTCCCAGGC-3′, Promega, Madison, WI) containing the NF-κB-binding motif, which was radiolabeled as described in our previous study [19]. Nuclear extracts were incubated with gel shift binding buffer (10 mM HEPES [pH 7.9], 50 mM KCl, 0.2 mM EDTA, 2.5 mM DTT, 10% glycerol, 0.05% NP-40, 0.25 mg/mL poly dI/poly dC, and protease inhibitor cocktail) for 10 min at room temperature. The mixture was then incubated with a ^32^P-labeled probe for 20 min at room temperature. DNA–protein complexes were resolved in a pre-electrophoresed 6% nondenaturing polyacrylamide gel at 4°C. Gels were dried and revealed by autoradiography.

### Western Blot Analysis

After treatment with the MWCNT samples, cells were harvested, washed twice with ice-cold PBS, and lysed in modified RIPA buffer with protease inhibitors. The cell lysates were cleared by centrifugation at 12,000 × g for 30 min at 4°C. The total protein content of the resulting supernatant was collected and quantified by the Bradford method. For western blot analysis, equal amounts of total protein were loaded onto 12% SDS-polyacrylamide gels and electrophoretically transferred onto a PVDF membrane (Millipore, Bedford, MA). After blocking, the membranes were then incubated overnight at 4°C with specific primary antibodies. Antibodies against poly ADP-ribose polymerase (PARP), Bax, β-actin, cytochrome c, p47^phox^, p67^phox^, and p65 were obtained from Santa Cruz Biotechnology (Cruz, CA). Antibodies against pro-caspase-3, cleaved caspase-3, pro-caspase-8, cleaved caspase-8, pro-caspase-9, cleaved caspase-9, PARP, phospho-JNK, phospho-p38, and phospho-ERK1/2 were obtained from New England Biolabs (Beverly, MA, USA). After washing three times with TBST, the membranes were incubated with horseradish peroxidase (HRP)-conjugated secondary antibody for 1 h. The blots were developed using enhanced chemiluminescence (ECL) according to the manufacturer’s protocol (Amersham Biosciences, Piscataway, NJ, USA).

### Statistical Analysis

Results are presented as the means ± standard deviation (SD) of the triplicate experiments. Comparisons between groups were evaluated by two-side Student’s *t* test or one-way ANOVA. A difference was considered significant at *p*<0.05.

## Results

### Characterization of the MWCNTs-COOH and MWCNTs-PEG

**Figure 1:**
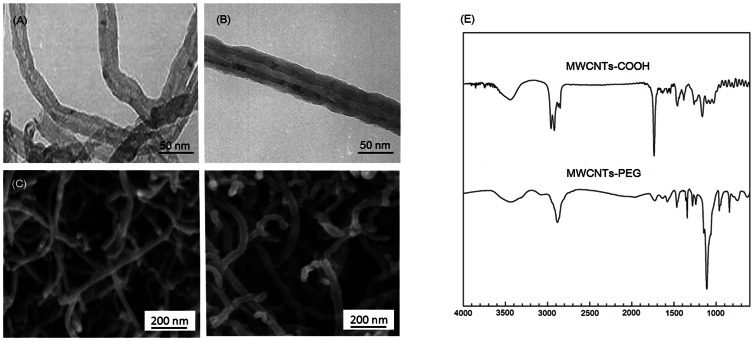
Representative TEM and SEM images of (A, C) MWCNTs-COOH and (B, D) MWCNTs-PEG. **(E) FT-IR spectra of MWCNTs-COOH and MWCNTs-PEG.**

TEM images (Figure 1A, B) demonstrated that both of the *f*-MWCNT samples were largely free from amorphous carbon and catalytic metals. SEM images (Figure 1C, D) showed a uniform surface coating of PEG on MWCNTs after functionalization, which resulted in an increase of the average diameter of the MWCNTs-PEG to 27.3±12.5 nm (Table 1). XPS analysis suggested that the surface of the MWCNTs was significantly modified with carboxylic acid groups (-COOH) after acid treatment, as well as successively functionalized with PEG (Table S1). The chemical compositions of MWCNTs-COOH and MWCNTs-PEG were further confirmed by FT-IR spectra (Figure 1E). TGA analysis showed that the amount of carboxylic acid groups and PEG bound to the MWCNTs was about 11.3 wt% and 27.4 wt%, respectively. ICP-MS data showed that the iron impurity of MWCNTs-COOH and MWCNTs-PEG was less than 0.0074% and 0.0042% by weight, respectively (Table 1). The zeta potential of the MWCNTs-COOH and MWCNTs-PEG in RPMI1640 medium was −20.2 mV and −35.6 mV, respectively, indicating that MWCNTs-PEG are possibly more stable in medium compared with the MWCNTs-COOH.

**Table 1:** Characterization of MWCNTs-COOH and MWCNTs-PEG.

	Length*	Diameter*	Purity	Surface	Surface
	(µm)	(nm)	(wt% Fe)	potential(mV)	area (m^2^/g)
MWCNTs-COOH	0.9±0.5	24.6±9.7	0.0074	−20.2	98
MWCNTs-PEG	0.8±0.6	27.3±12.5	0.0042	−35.6	78
Method	TEM	TEM	ICP-MS	Zeta potential	BET

* Average diameters and lengths of MWCNTs-COOH and MWCNTs-PEG were determined by TEM images (n = 20).

### Cytotoxic Effects of MWCNTs-COOH and MWCNTs-PEG on Macrophages

**Figure 2:**
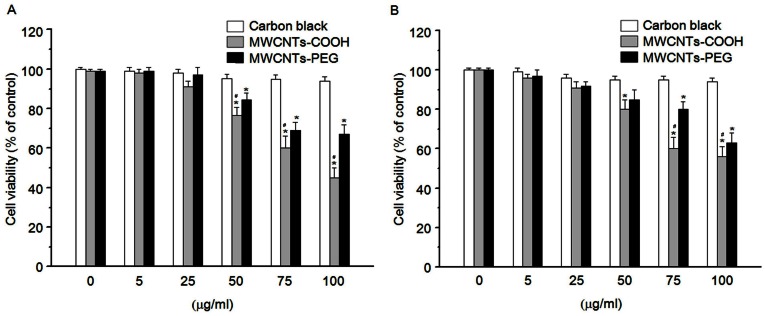
Cytotoxic effects of MWCNTs-COOH and MWCNTs-PEG on macrophages. (A) RAW 264.7 cells and (B) primary rat peritoneal macrophages were incubated with or without indicated concentrations of *f*-MWCNTs samples for 24 h. At the end of the incubation period, the WST-1 assay was performed to evaluate the cytotoxicity. Data are representative of three independent experiments and are expressed as the mean ± SD of at least three experiments. *p<0.05 compared to control sample, #*p*<0.05 compared to MWCNTs-PEG.

In a primary screening test, we examined whether MWCNTs-COOH or MWCNTs-PEG were cytotoxic. RAW 264.7 cells were treated with various concentrations of MWCNTs-COOH and MWCNTs-PEG for 24 h, and cell viability was determined by the WST-1 assay. As shown in Figure 2A and B, both MWCNTs-COOH and MWCNTs-PEG at the concentration of 75 µg/mL significantly reduced the viability of RAW 264.7 cells after 24 h of incubation. MWCNTs-PEG was found to be less toxic than MWCNTs-COOH at the same concentration. Similarly, using primary rat peritoneal macrophages, MWCNTs-PEG was also found to be less toxic effect than MWCNTs-COOH. Carbon black is usually used as a negative control and exerted a very minimal effect against RAW 264.7 cells and peritoneal macrophages (Figure 2A, B).

### Cellular Uptake and Distribution of MWCNTs-COOH and MWCNTs-PEG

**Figure 3:**
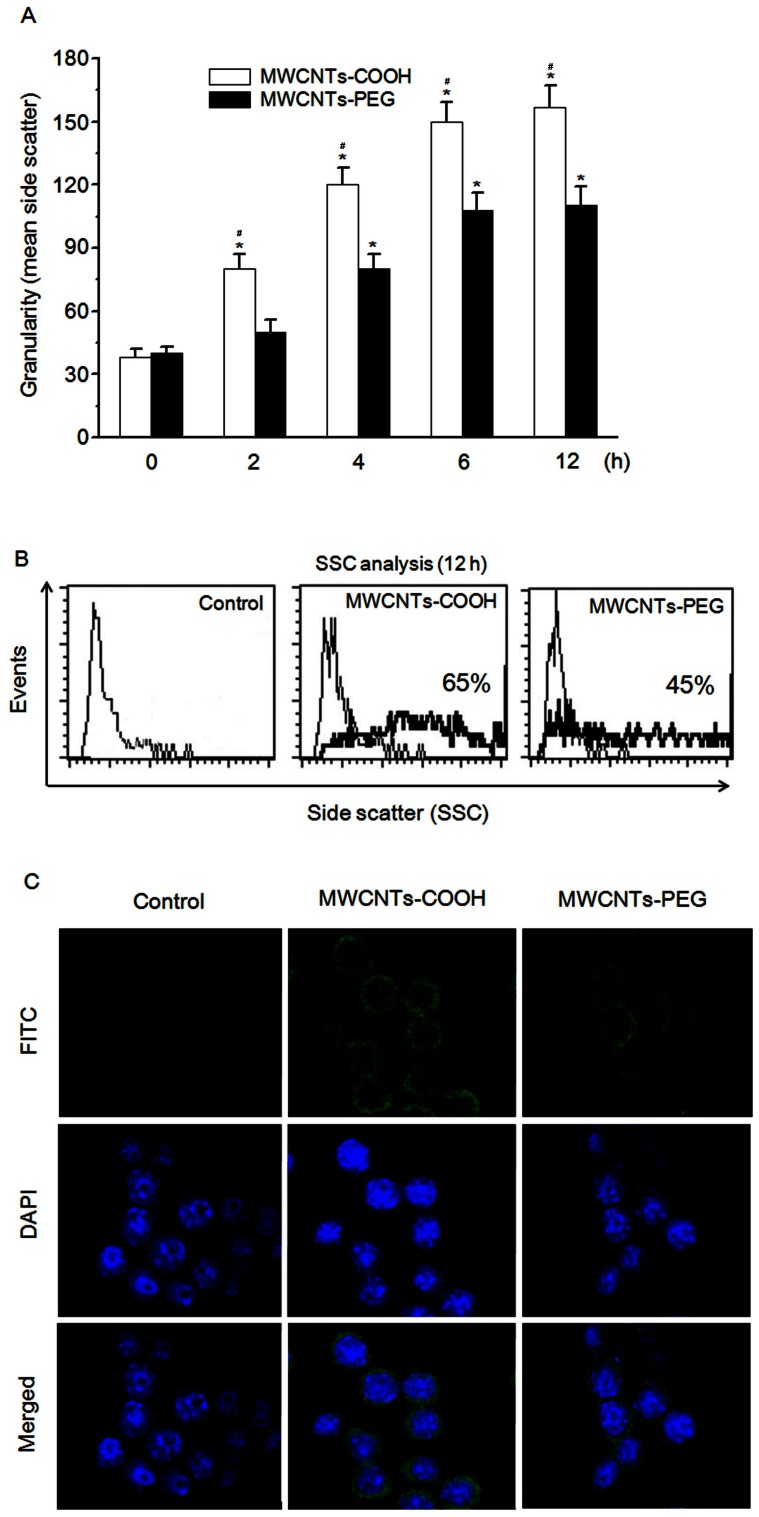
Cellular uptake and distribution of MWCNTs-COOH and MWCNTs-PEG. (A) Quantitative analysis of cellular uptake of MWCNTs-COOH and MWCNTs-PEG by RAW 264.7 cells as shown by flow cytometry using the light SSC parameter. Cells were treated with or without *f*-MWCNT samples for the indicated time and assayed for SSC intensity by flow cytometry. Data are representative of three independent experiments and are expressed as the mean ± SD of at least three experiments. *p<0.05 compared to control sample, #*p*<0.05 compared to MWCNTs-PEG. (B) Intracellular distribution of MWCNTs-COOH and MWCNTs-PEG in RAW 264.7 cells viewed under a fluorescence microscope. Cells were incubated with or without FITC-labeled *f*-MWCNTs samples for 12 h, and then processed for CLSM examination.

It has been proposed that the intracellular concentration of nanoparticles is reflected in the intensity of light SSC measured by flow cytometry [21]. Using this experimental approach, a time-dependent increase in the light SSC of cells was detected after treatment with MWCNTs-COOH, which began as early as 2 h (Figure 3A, B). In contrast, MWCNTs-PEG showed less intensity of light SSC following treatment (Figure 3A, B) compared with MWCNTs-COOH. Next, we studied the intracellular distribution of the *f*-MWCNTs in RAW 264.7 cells. Confocal laser scanning microscopy (CLSM) analysis using FITC-labeled *f*-MWCNTs revealed that the fluorescence signals were located mainly in the cytoplasm within 12 h of incubation, indicating the lack of *f*-MWCNTs translocating to the nucleus. Consistent with the intensity of SSC, MWCNTs-COOH showed stronger fluorescence compared with MWCNTs-PEG. The cell uptake and distribution of *f*-MWCNTs was directly confirmed using optical microscopy (Figure S1).

### Induction of Apoptosis by MWCNTs-COOH and MWCNTs-PEG

**Figure 4:**
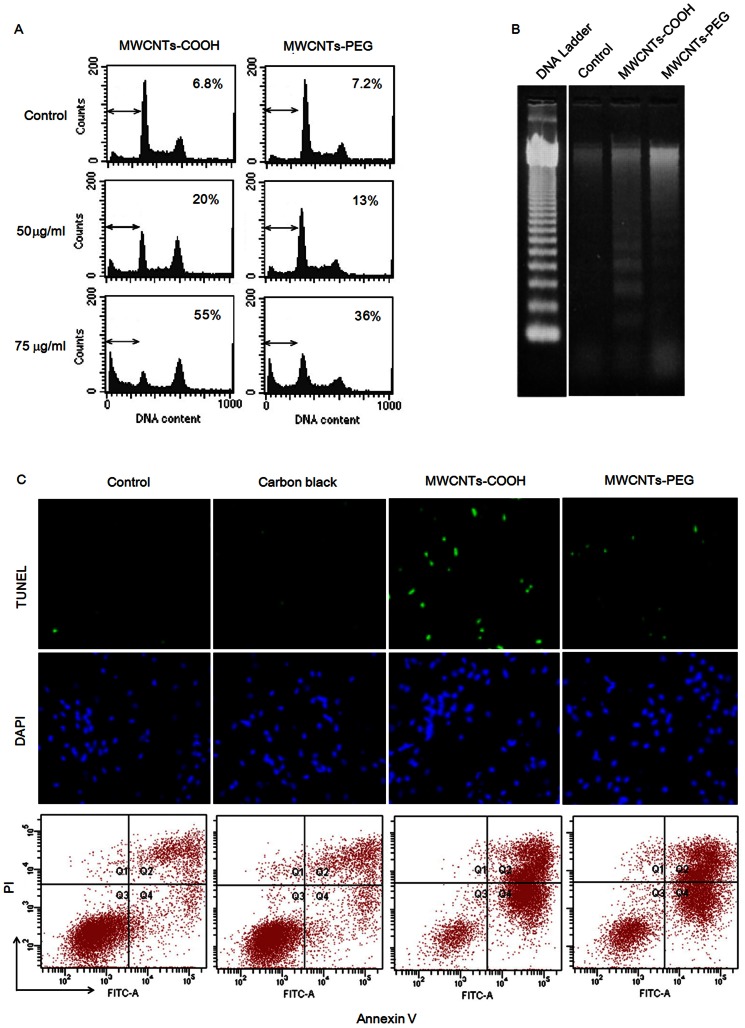
MWCNTs-COOH and MWCNTs-PEG induced apoptosis of RAW 264.7 cells. Cells were treated with or without either MWCNTs-COOH or MWCNTs-PEG for 24 h, and apoptosis was evaluated by (A) flow cytometry using propidium iodide, (B) gel electrophoretic DNA fragmentation analysis, (C) TUNEL assay, and annexin-V-FLUOS labeling assay. Representative images from three independent experiments are shown.

To investigate whether macrophages undergo apoptosis in response to treatment with either MWCNTs-COOH or MWCNTs-PEG, RAW 264.7 cells were exposed to various concentrations of the *f*-MWCNTs for 24 h. Apoptosis (sub-G1 group) was then examined by propidium iodide staining with flow cytometry. As shown in Figure 4A, both MWCNTs-COOH and MWCNTs-PEG induced apoptotic cell death in RAW 264.7 cells. However, the apoptotic cell death of RAW 264.7 cells induced by MWCNTs-COOH appeared to be more pronounced as compared with MWCNTs-PEG. The apoptosis-inducing effect of MWCNTs-COOH and MWCNTs-PEG was further confirmed by DNA fragmentation, TUNEL assay, and annexin-V-FLUOS labeling assay (Figure 4B, C). These results indicated that the cytotoxicity elicited by MWCNTs-COOH and MWCNTs-PEG may manifest as apoptotic cell death.

### MWCNTs-COOH and MWCNTs-PEG Induced the Activation of caspase- 3 and -9, and Loss of Mitochondrial Membrane Potential (Δ*Ψ*m)

**Figure 5:**
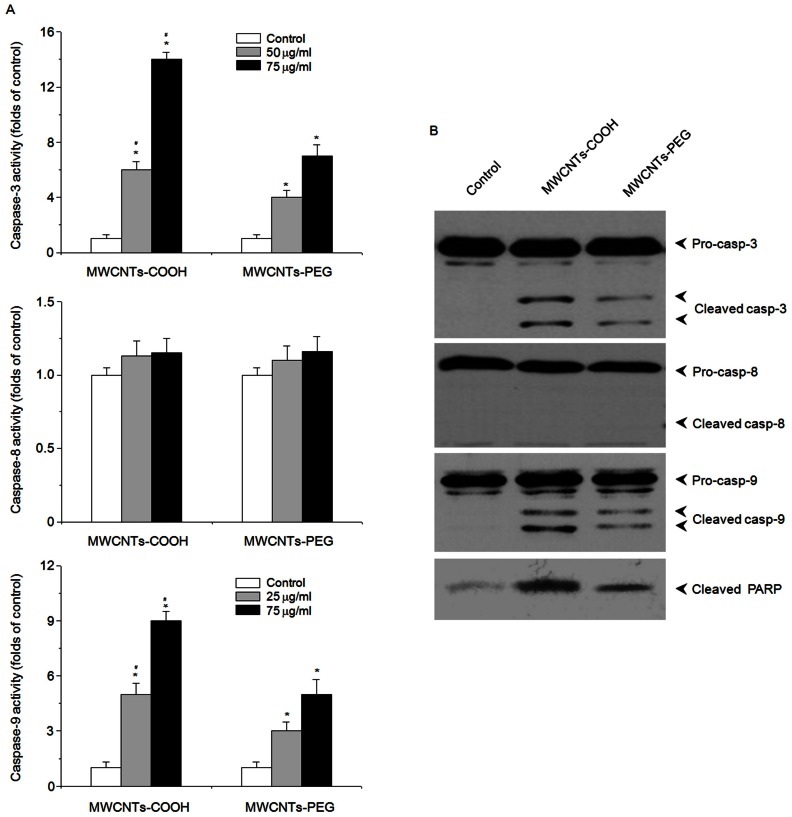
MWCNTs-COOH and MWCNTs-PEG induced the activation of caspase-3 and −9 in RAW 264.7 cells. Cells were treated with or without either MWCNTs-COOH or MWCNTs-PEG for 24 h. The activation of caspase-3, −8, −9 and PARP was measured by (A) caspase activity or (B) western blot analysis. Data are representative of three independent experiments and are expressed as the mean ± SD of at least three experiments. *p<0.05 compared to control sample, #*p*<0.05 compared to MWCNTs-PEG. Representative images of the activation of caspase-3, −8, −9, and PARP by western blot analysis are shown.

To gain further insights into the molecular events associated with apoptosis of macrophages induced by MWCNTs-COOH and MWCNTs-PEG, we analyzed caspases-3, −8, and −9 activities in RAW 264.7 cells using a caspase fluorogenic peptide substrate kit. Treatment of RAW 264.7 cells with MWCNTs-COOH led to a dose-dependent increase in activation of caspase-3 and -9, but not caspases-8 (Figure 5A). In contrast, MWCNTs-PEG induced less activation of caspase-3 and −9 in macrophages as compared with MWCNTs-COOH. The activation of caspases-3 and −9 was verified by western blot analysis (Figure 5B). In addition, the cleavage of PARP, an intracellular substrate of caspase-3, showed similar results (Figure 5B).

**Figure 6:**
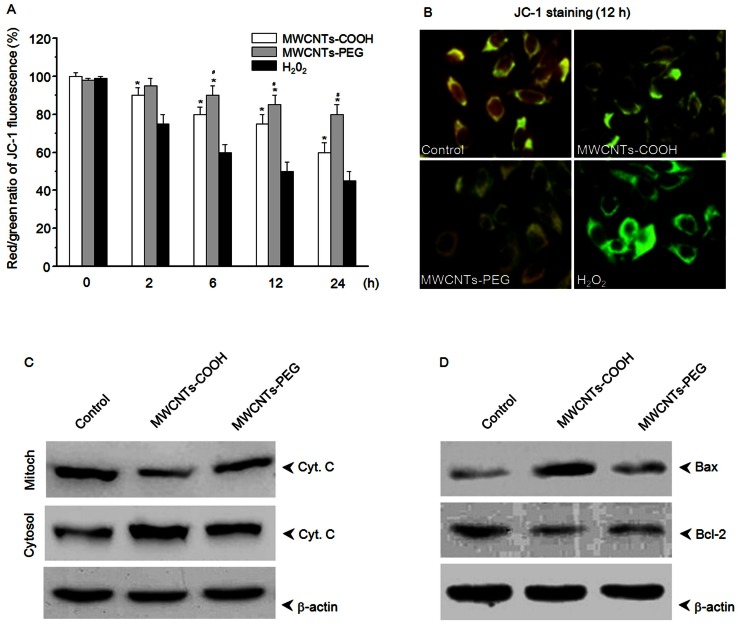
MWCNTs-COOH and MWCNTs-PEG induced a mitochondria-associated death pathway in RAW 264.7 cells. (A, B) MWCNTs-COOH and MWCNTs-PEG induced the loss of Δ*Ψ*m in RAW 264.7 cells. Cells treated with or without 75 µg/mL of MWCNT samples for the indicated times were stained with the mitochondria-selective JC-1 dye, and then analyzed by flow cytometry and fluorescence microscope. Data are representative of three independent experiments and are expressed as the mean ± SD of at least three experiments. *p<0.05 compared to control sample, #*p*<0.05 compared to MWCNTs-PEG. (C) Western blot analysis of the effects of MWCNTs-COOH and MWCNTs-PEG on translocation of cyto c, and expression of Bax and Bcl-2 in RAW 264.7 cells. Cells were treated with or without 75 µg/mL of MWCNT samples for 24 h. Data represent similar results from three independent experiments.

Using JC-1 as a marker of Δ*Ψ*m, flow cytometry revealed that MWCNTs-COOH depolarized the level of Δ*Ψ*m in RAW 264.7 cells. The response occurred in a time-dependent manner, and a significant depletion of Δ*Ψ*m could be detected within 2 h of treatment (Figure 6A, B). Furthermore, western blot analysis showed that MWCNTs-COOH induced the release of cytochrome c from the mitochondria to the cytosol, suppressed the expression of pro-survival Bcl-2 proteins (such as Bcl-2), and increased the expression of pro-apoptosis Bcl-2 proteins (such as Bax) (Figure 6C, D). In contrast, MWCNTs-PEG had less effect on mitochondria-associated apoptotic components (Figure 6A-D).

### Generation of ROS and Activation of NADPH Oxidase

**Figure 7:**
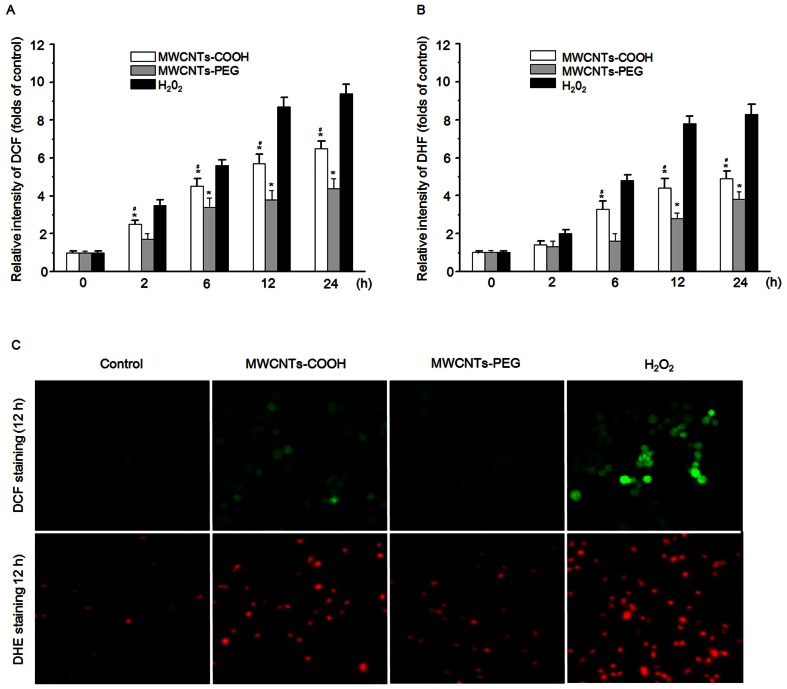
MWCNTs-COOH and MWCNTs-PEG induced ROS generation in RAW 264.7 cells. Cells were treated with or without 75 µg/mL of MWCNT samples for the indicated times, and the ROS levels were measured by (A) DCF staining and (B) HE staining using a flow cytometer or (C) fluorescence microscope. Data are representative of three independent experiments and are expressed as the mean ± SD of at least three experiments. Hydrogen peroxide (H_2_O_2_) (1 mM) was used as the positive control. *p<0.05 compared to control sample, #*p*<0.05 compared to MWCNTs-PEG. Data represent similar results from three independent experiments.

To elucidate whether intracellular accumulation of ROS contributes to apoptosis of macrophages induced by MWCNTs-COOH and MWCNTs-PEG, the fluorophore H_2_DCF-DA was used to detect ROS generation following exposure of RAW 264.7 cells to 75 µg/mL of either MWCNTs-COOH or MWCNTs-PEG for up to 24 h. As shown in Figure 7A and C, MWCNTs-PEG showed less induction of ROS generation as compared with MWCNTs-PEG after 12 h of incubation. To specify the nature of ROS, the superoxide-specific dye DHE was used to detect superoxide generation. As observed with DCF staining, treatment with MWCNTs-PEG induced much less accumulation of superoxide radicals than MWCNTs-COOH (Figure 7B, C).

**Figure 8:**
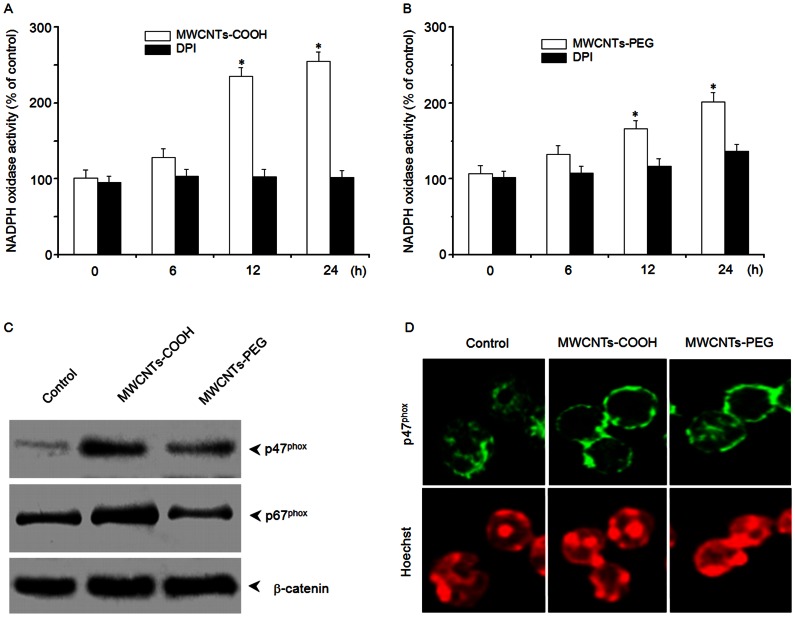
Effects of MWCNTs-COOH and MWCNTs-PEG on NADPH oxidase activity. RAW 264.7 cells were treated with or without 75 µg/mL of either (A) MWCNTs-COOH or (B) MWCNTs-PEG for up to 12 h, and NADPH oxidase was measured. Data are representative of three independent experiments and are expressed as the mean ± SD of at least three experiments. *p<0.05 compared to control sample. (C) MWCNTs-COOH and MWCNTs-PEG stimulated membrane translocation of p47^phox^ and p67^phox^. After treatment of RAW 264.7 cells with or without MWCNT samples for 12 h, cell lysates were isolated from the membrane fraction. Western blot analysis was used to detect p47^phox^ and p67^phox^. Blots are representative of at least three independent experiments. (D) Immunolocalization of p47^phox^ after exposure of RAW 264.7 cells to 75 µg/mL of MWCNT samples for 12 h. Images are representative of three independent experiments.

Because uptake of particles via phagocytosis can lead to ROS generation by activating the membrane-bound NADPH oxidase, we measured the NADPH oxidase activity to determine whether it was involved in ROS accumulation in macrophages treated with either MWCNTs-COOH or MWCNTs-PEG. As shown in Figure 8A and B, treatment of RAW 264.7 cells with MWCNTs-COOH elicited higher NADPH oxidase activity than treatment with MWCNTs-PEG. Activation of NADPH oxidase requires the translocation of the cytosolic components p47^phox^ and p67^phox^ to the cell membrane. Therefore, we further examined the effects of MWCNTs-COOH and MWCNTs-PEG on membrane translocation of p47^phox^ and p67^phox^ proteins by western blot analysis. Figure 8C shows that MWCNTs-COOH induced more membrane translocation of p47^phox^ and p67^phox^ than MWCNTs-PEG, which was further confirmed by immunolocalization of anti-p47^phox^ antibody (Figure 8D).

### Activation of MAPK and NF-κB Pathways

**Figure 9:**
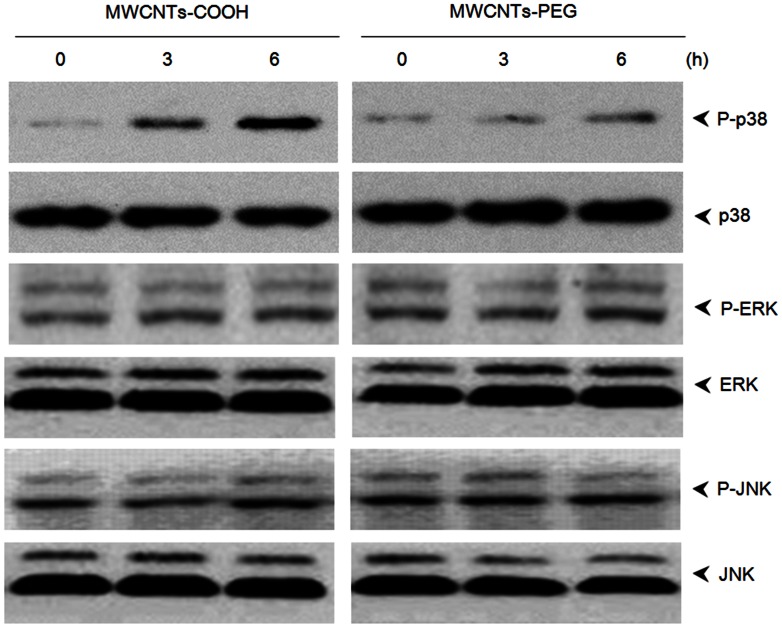
Effects of MWCNTs-COOH and MWCNTs-PEG on the MAPKs pathway. RAW264.7 cells were treated with 75 µg/mL of either MWCNTs-COOH or MWCNTs-PEG for the indicated times. The whole-cell lysate was analyzed by western blot analysis for phosphorylated and nonphosphorylated p38, ERK, and JNK. Data represent similar results from three independent experiments.

**Figure 10:**
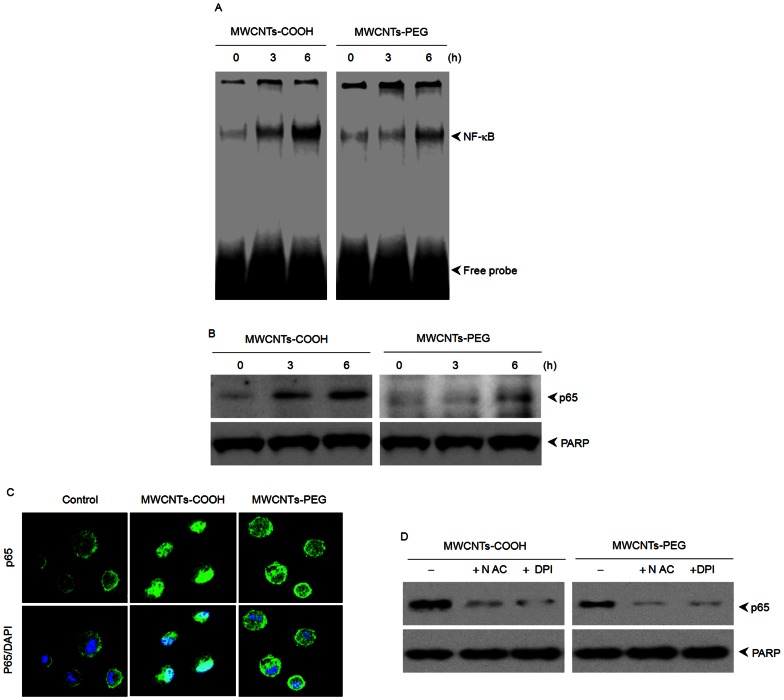
Effects of MWCNTs-COOH and MWCNTs-PEG on NF-κB activation. RAW264.7 cells were treated with 75 µg/mL of either MWCNTs-COOH or MWCNTs-PEG for the indicated times, then EMSA assay and western blot analysis were used to determine (A) NF-κB DNA-binding activity and (B) levels of cytoplasmic IκBα and nuclear p65, respectively. (C) Translocation of p65 from cytosol to nuclei. RAW264.7 cells were treated with or without 75 µg/mL of MWCNT samples for 6 h, and p65 was then immunofluorescently stained (green). (D) Pretreatment of RAW264.7 cells with ROS scavenger (NAC, DPI) inhibited NF-κB activation. Data represent similar results from three independent experiments.

MAPKs and NF-κB are key redox-sensitive intercellular mediators of apoptotic signaling. To determine the effects of MWCNTs-COOH and MWCNTs-PEG on MAPK activation, we studied the phosphorylation of three types of MAPKs: p38 MAPK, ERK, and JNK. MWCNTs-COOH induced higher activation of p38 MAPK than MWCNTs-PEG; however, both MWCNTs-COOH and MWCNTs-PEG had little effect on either JNK or ERK activity **(** Figure 9 **)**. As illustrated in Figure 10A, compared with MWCNTs-COOH, treatment with MWCNTs-PEG induced much less NF-κB DNA-binding activity, as measured by EMSA assay after 3 h of treatment. Consistent with the EMSA data, western blot analysis revealed that MWCNTs-PEG caused less degradation of IκBα and nuclear translocation of p65 than MWCNTs-COOH (Figure 10B). The nuclear translocation of p65 was confirmed by immunolocalization of anti-p65 antibody (Figure 10C).

**Figure 11:**
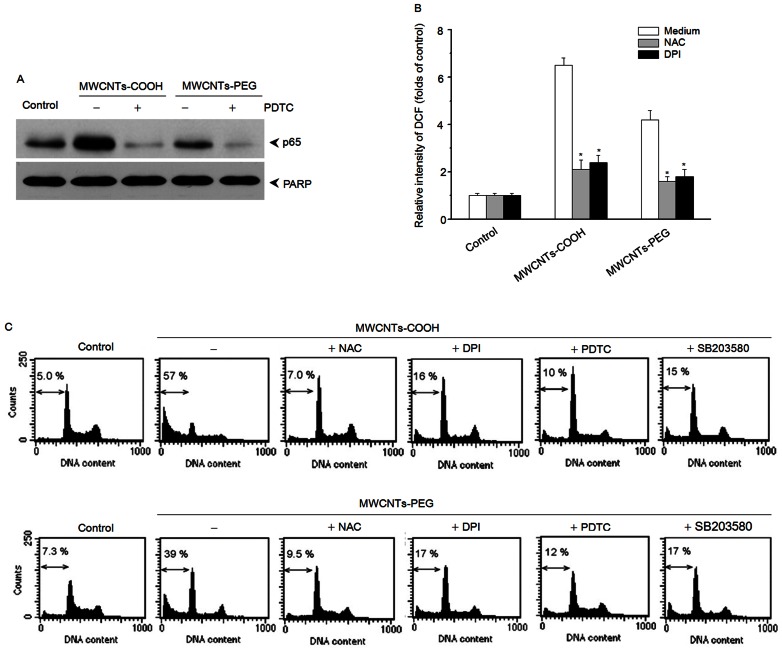
Involvement of NADPH oxidase, ROS, p38 MAPK, and NF-κB in MWCNTs-COOH- and MWCNTs-PEG-induced apoptosis of RAW264.7 cells. Cells were pretreated with a ROS scavenger (NAC), NADPH oxidase inhibitor (DPI), p38 inhibitor (SB203580), or NF-κB inhibitor (PDTC) for 1 h, and then treated with either MWCNTs-COOH or MWCNTs-PEG for 24 h. Western blot analysis and DCF staining using flow cytometry were used to determine levels of cytoplasmic p65 (A) and intracellular ROS (B), then the apoptosis of RAW264.7 cells were evaluated by flow cytometry using PI staining. Data are representative of three independent experiments (C). *p<0.05 compared to control sample.

To determine the causal relationship between apoptosis and ROS, p38 MAPK, or NF-κB RAW 264.7 cells were treated with either MWCNTs-COOH or MWCNTs-PEG for 24 h with or without addition of an inhibitor of ROS (NAC), NADPH oxidase (DPI), NF-κB (PDTC), or p38 MAPK (SB203580). Figure 11A and B demonstrates that pretreatment of RAW264.7 cells with NAC, DPI, or PDTC inhibited ROS accumulation and NF-κB activation. As shown in Figure 11C, these specific inhibitors significantly suppressed apoptosis induced by MWCNTs-COOH and MWCNTs-PEG. These findings indicated that MWCNTs-COOH- and MWCNTs-PEG-induced apoptosis depended in part on NADPH oxidase-driven ROS, p38 MAPK, and NF-κB pathways.

## Discussion

In this study, we elucidated the role of surface functionalization in the apoptotic effect of *f*-MWCNTs using RAW 264.7 cells as a cellular model. Our goal was to obtain basic information for use in the development of safe and effective nanomaterials. We demonstrated that MWCNTs-COOH were significantly more cytotoxic and associated with significantly higher apoptotic cell death than MWCNTs-PEG *in vitro*. The difference in apoptotic response may be attributed to the differences in cell uptake of *f*-MWCNTs by macrophages, as well as the activation of the oxidative stress-responsive apoptotic pathway. The present study provides new insights into the molecular basis of the biological effects of the surface characteristics of MWCNTs.

Our results showed that treatment with MWCNTs-PEG was less cytotoxic than MWCNTs-COOH to macrophages (Figure 2A, B). The difference in the cytotoxic effect between MWCNTs-COOH and MWCNTs-PEG is most likely due to their differences in surface chemistry. Consistent with our thinking, the results obtained by other studies indicated that surface-functionalized CNTs induce less toxic effects than unmodified ones [12]–[15], [17]. Previous studies have demonstrated the important role of the surface characteristics of nanoparticles in cellular uptake, bioactivity, and toxic responses [8]. Therefore, we speculated that the less cytotoxic effect of the MWCNTs-PEG compared with the MWCNTs-COOH might result from a difference in the cellular uptake between them. Data obtained from flow cytometric SSC analysis showed the difference in the amounts of MWCNTs-COOH or MWCNT-PEGs that were internalized by macrophages. Both types of *f*-MWCNTs accumulated within the cytoplasm, as verified by CLSM analysis using FITC-labeled MWCNTs and light microscopy. The cellular uptake data correlated well with the cytotoxicity results, suggesting that the abundance of cell uptake of *f*-MWCNTs plays a role in their cytotoxic effect [18], [28]. Currently, the mechanisms involved in the cellular uptake of MWCNTs-COOH and MWCNTs-PEG are still unclear. Recent reports have shown that cellular uptake of MWCNTs is positively correlated with their surface charge, and negatively-charged MWCNTs-COOH may facilitate transport of MWCNTs through the cell membrane [18]. In contrast, the nanostructures of PEG-modified CNTs were expected to have less interaction with the cellular membranes due to the formation of a hydrophilic stealth coating, leading to reduced cellular uptake [17]. Furthermore, hydrophilic PEG polymers can interfere with the formation of protein corona as well as particle opsonization, a mechanism in which opsonins play a key role in nanoparticle uptake in macrophages [11]. Similar findings have been reported for other PEG-modified nanoparticles, such as quantum dots [29], and super paramagnetic magnetite nanoparticles [30]. Further investigation is warranted to compare the mechanism of cellular uptake and intracellular trafficking of different types of *f*-MWCNTs.

Previous studies have demonstrated that nanomaterials can induce apoptosis, which may be considered an indicator of toxicity for many nanomaterials [19]. Therefore, we proposed that the cytotoxic effects of MWCNTs-COOH and MWCNTs-PEG may be attributed to their ability of inducing apoptosis of macrophages. Here, our results showed that either MWCNTs-COOH or MWCNTs-PEG at the concentration of 50 µg/mL induced apoptosis of RAW264.7 cells, as revealed by DNA fragmentation, TUNEL assay, and translocation of phosphatidylserine (Figure 3A, B). However, MWCNTs-PEG showed significantly lower induction of apoptotic cell death compared with MWCNTs-COOH. These results were confirmed by the activation of caspase-3 and -9, the expression of pro-apoptotic Bax protein and pro-survival Bcl-2 protein, and translocation of cytochrome c from mitochondria to cytosol. These observations correlated well with previous results that suggested that caspase-dependent mechanisms with intrinsic pathways were involved in apoptosis induced by MWCNTs [31]–[33]. This demonstrated the role of surface functionalization in the apoptotic effect.

The production of ROS by nanomaterials is generally considered a major contributor to their toxicity [34]. Uptake of nanomaterials via phagocytosis has been demonstrated to trigger an activation of the membrane-bound NADPH oxidase, which catalyzes the conversion of oxygen to superoxide radicals [34]. Here, our results showed that MWCNTs-PEG induced less production of ROS and superoxide radicals, involving less activation of NADPH oxidase as compared with MWCNTs-COOH in microphages (Figure 7). These results are in line with recent findings, which showed that PEG-modified CNTs significantly decreases ROS-mediated toxicological response *in vitro* [16]–[18]. They also further supported the previously described critical role of NADPH oxidase in the generation of ROS in response to CNTs [21].

ROS have been demonstrated to interfere with redox-sensitive signaling pathways, in particular with a series of mitogen activated protein kinases (MAPKs), including p38 MAPK, c-Jun-N-terminal kinases (JNK), and extracellular signal-related kinases (ERK1/2) [35]. Here, our results showed that MWCNTs-PEG evoked less activation of p38 MAPK than MWCNTs-COOH. However, both MWCNTs-COOH and MWCNTs-PEG did not activate either JNK or ERK1/2 pathways, suggesting that the cytotoxic effects induced by *f*-MWCNTs might be dependent on p38 MAPK, but independent of ERK and JNK signaling. These results are consistent with previous studies that have shown that ROS-mediated activation of p38 MAPK cascades are critical to the adverse effects observed upon exposure to MWCNTs [36], [37]. The MAPK signaling pathway has been shown to play a role in NF-κB activation, leading to the induction of early response genes that are critical in apoptosis [35]. In this study, we found that MWCNTs-PEG showed less activation of NF-κB than MWCNTs-COOH. This suggests that the less apoptotic effect of MWCNTs-PEG may result from less activation of MAPKs and NF-κB. To further elucidate whether NADPH-derived ROS, p38 MAPK, or NF-κB participates in the apoptotic death of macrophages, RAW 264.7 cells were treated with either MWCNTs-COOH or MWCNTs-PEG in the presence or absence of their specific inhibitors. Our results showed that MWCNTs-COOH- or MWCNTs-PEG-induced apoptosis was significantly blocked by addition of DPI, NAC, PDTC, or SB253580. These data strongly suggest that either MWCNTs-COOH- or MWCNTs-PEG-induced apoptosis is associated with the p38 MAPK and NF-κB pathways.

### Conclusions

We demonstrated the role of surface characteristics in the cytotoxic and apoptotic effects of *f*-MWCNT on macrophages, and shed light on their underlying mechanisms of action. Our data showed that MWCNTs-PEG induces less apoptotic cell death of macrophages compared with MWCNTs-COOH. The mechanism of these effects involves differences in cellular uptake of *f*-MWCNTs, NADPH oxidase activation, as well as oxidative stress-responsive pathways. However, the current understanding of the molecular basis of altering biocompatibility of *f*-MWCNTs *in vitro* is not sufficient to reflect the real biological profile *in vivo.* We expect that further studies of the relationship between surface characteristics and biological effects will provide useful information for nanomaterial designs or applications that reduce harmful impact on humans and the environmental.

## Supporting Information

Figure S1 **Cellular uptake of MWCNTs-COOH and MWCNTs-PEG by RAW 264.7 cells examined under a light microscope.**
(DOC)

Table S1 **XPS analysis of MWCNTs-COOH and MWCNTs-PEG.**
(DOC)
