# Supplementary material for: Modulation of Apoptotic Pathways of Macrophages by Surface-Functionalized Multi-Walled Carbon Nanotubes
Source: PLoS One. 2013 Jun 6;8(6):e65756. doi: 10.1371/journal.pone.0065756 (PMC3675050; doi:10.1371/journal.pone.0065756)
Supplement: Figure S1 — Cellular uptake of MWCNTs-COOH and MWCNTs-PEG by RAW 264.7 cells examined under a light microscope. (DOC) [file pone.0065756.s001.doc]

**Supplementary information**

**Figure S1.**


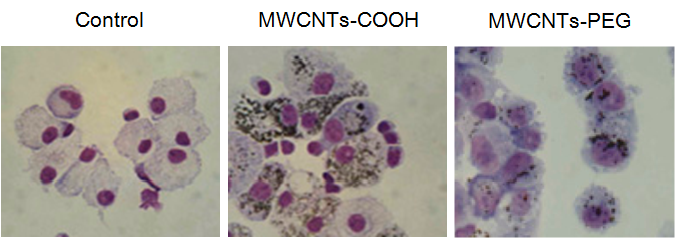


Cellular uptake of MWCNTs-COOH and MWCNTs-PEG by RAW 264.7 cells examined under a light microscope. After treatment, slides were fixed in methanol (100%; 1 min) and stained using the Diff-Quik Staining kit (Beyotime Biotech, Haimen, China) before observation by light microscopy (100 × magnification). The images clearly showed that the cell uptake of MWCNTs-COOH was significantly higher than that of MWCNTs-PEG.
