# Supplementary material for: Modulation of Apoptotic Pathways of Macrophages by Surface-Functionalized Multi-Walled Carbon Nanotubes
Source: PLoS One. 2013 Jun 6;8(6):e65756. doi: 10.1371/journal.pone.0065756 (PMC3675050; doi:10.1371/journal.pone.0065756)
Supplement: Table S1 — XPS analysis of MWCNTs-COOH and MWCNTs-PEG. (DOC) [file pone.0065756.s002.doc]

Table S1. XPS analysis of MWCNTs-COOH and MWCNTs-PEG.

|  | MWCNTs-COOH |  | MWCNTs-PEG |  |
| --- | --- | --- | --- | --- |
| Peak | Position (eV) | Atomic conc. (%) | Position (eV) | Atomic conc. (%) |
| C1s | 284.54 | 71.45 | 284.56 | 69.76 |
| -C-OH | 286.77 | 9.82 | 285.89 | 17.25 |
| -C=O | 286.48 | 6.23 | 286.68 | 6.78 |
| -COOH | 288.78 | 12.50 | 288.78 | 6.21 |

XPS analysis indicated that the functional groups on the surface of the MWCNTs were changed after acid treatment and chemical functionalization with PEG. These observations suggested that the surface of the MWCNTs was significantly modified with carboxylic acid groups (-COOH) after acid treatment, as well as successively functionalized with PEG.
